# Thermal transport crossover from crystalline to partial-crystalline partial-liquid state

**DOI:** 10.1038/s41467-018-07027-x

**Published:** 2018-11-09

**Authors:** Yanguang Zhou, Shiyun Xiong, Xiaoliang Zhang, Sebastian Volz, Ming Hu

**Affiliations:** 1 Aachen Institute for Advanced Study in Computational Engineering Science (AICES), RWTH Aachen University, Aachen, 52062 Germany; 2 Department of Mechanical and Aerospace Engineering, University of California Los Angeles, Los Angeles, CA 90095 USA; 3 Functional Nano and Soft Materials Laboratory (FUNSOM) and Collaborative Innovation Center of Suzhou Nano Science and Technology, Soochow University, Suzhou, 215123 China; 4 Joint International Research Laboratory of Carbon-Based Functional Materials and Devices, Soochow University, Suzhou, 215123 China; 5 Key Laboratory of Ocean Energy Utilization and Energy Conservation of Ministry of Education, School of Energy and Power Engineering, Dalian University of Technology, Dalian, 116024 China; 6 CNRS, UPR 288 Laboratoire d’Energétique Moléculaire et Macroscopique, Combustion (EM2C), Ecole Centrale Paris, Grande Voie des Vignes, 92295 Châtenay-Malabry, France; 7 LIMMS/CNRS-IIS(UMI2820) Institute of Industrial Science, University of Tokyo 4-6-1 Komaba, Meguro-ku Tokyo, 153-8505 Japan; 8 Institute of Mineral Engineering, Faculty of Geosciences and Materials Engineering, RWTH Aachen University, Aachen, 52064 Germany; 9 Department of Mechanical Engineering, University of South Carolina, Columbia, SC 29208 USA

## Abstract

Phase-change materials (crystalline at low temperatures and partial-crystalline partial-liquid state at high temperatures) are widely used as thermoelectric converters and battery electrodes. Here, we report the underlying mechanisms driving the thermal transport of the liquid component, and the thermal conductivity contributions from phonons, vibrations with extremely short mean free path, liquid and lattice-liquid interactions in phase-changed Li_2_S. In the crystalline state (*T* ≤ 1000 K), the temperature dependent thermal conductivity manifests two different behaviors, i.e., a typical trend of 1/*T* below 800 K and an even faster decrease between 800 and 1000 K. For the partial-crystalline partial-liquid Li_2_S when *T* ≥ 1100 K, the contributions of liquid and lattice-liquid interactions increase significantly due to the fluidization of Li ions, and the vibrations with extremely short mean free path, presumably assimilated to diffusons, can contribute up to 46% of the total thermal conductivity at *T* = 1300 K.

## Abstract (web summary)

Phase-change materials are applied as thermoelectric converters and battery electrodes, but underlying mechanisms are not fully understood. Here, the authors comprehensively describe thermal transport mechanisms of lithium sulfide based on molecular dynamics and first-principles simulations.

## Introduction

Heat transfer in perfect crystals has been well-studied, and has been shown of great importance in many disciplines, such as thermoelectrics (TEs)^1,2^, phononic materials^3^, and thermal management^4^. However, thermal transport in partial-crystalline partial-liquid state (PCPLS) of phase-change materials (PCMs), which are potential candidates for TEs (e.g., superionic *α*-phase Cu_2_Se, Ag_2_Te)^5–8^ and for the most popular electrodes (e.g., superionic *α*-phase Li_2_S and LiSi)^9–11^, is still poorly understood on the fundamental level. For instance, how does the liquid component affect lattice transport and how large are the contributions from the convection part and the convection-lattice part? Is the convection-lattice interaction an impacting mechanism in contrast to other well-studied two-component systems, such as electron-phonon interaction^12^? On the practical level, detailed understanding of the thermal transport properties in PCPLS of PCMs is a key point to optimize its TE performance and to solve the overheat issues in Li batteries. Although phenomenological explanations such as rattling-like thermal damping^5^ and strong anharmonicity^6^ have been proposed to explain the extremely low thermal conductivity (TC) in PCMs, such as Cu_2_Se, the quantitative relation between TC and the liquid motion, and how phonon transport in the crystalline part is affected by the liquid motion is still lacking in literature.

In this paper, we quantitatively investigate the mechanisms of heat transport in PCMs by taking Li_2_S as a case study using the reactive force field (ReaxFF)^10^ Green-Kubo equilibrium molecular dynamics (GK-EMD)^13^, the first-principles-based Boltzmann transport equation (BTE)^14^, as well as the frequency domain direct decomposition method (FDDDM)^15,16^ or the Sääskilahti method^17^. Although, the phase transition of PCM Li_2_S is widely studied^18^, the thermal transport behaviors have never been explored. We find that there is a transition from pure phonon transport to phonon-convection interactions in two-components transport in Li_2_S. The contributions to TC from phonons, convection, and phonon-convection are quantitatively characterized. Simulation details can be found in Method section and Supplemental Materials.

## Results

### Diffusion coefficient

**Fig. 1:**
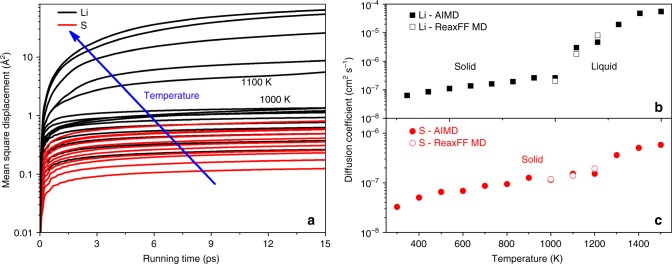
Diffusion coefficient calculations. **a** Mean square displacement and diffusion coefficient of **b** Li and **c** S at different temperatures obtained by ab initio molecular dynamics (AIMD) simulations and reactive force filed molecular dynamics (ReaxFF MD) simulations. The arrow indicates the increase of temperature

Figure 1 shows the diffusion coefficients obtained from ab initio molecular dynamics (AIMD) simulation and ReaxFF MD. It shows that the Li_2_S will change from pure solid or crystalline state (*β*-phase) to partial-crystalline partial-liquid state (superionic *α*-phase) as temperature arises, which is markedly different from the melting process^19–21^. The identified phase transition temperature falls between 1000 and 1100 K, which is in good accordance with previous AIMD (1050 K)^9^ and our ReaxFF MD results [Fig. 1], but higher than the experimental value of 900 K^22^. At low temperatures, the *β*-phase of Li_2_S possesses a fully ordered face centered cubic (FCC) structure with space group $Fm\bar 3m$. In all temperature ranges considered, S atoms have solid-like mobility while the mobility of Li ions is increased by more than one order of magnitude around 1100 K, indicating the fluidization of Li ions. When *T* ≥ 1100 K, atoms vibrate around their equilibrium positions while Li ions can hop into their neighbor sites. Within the *α*-Li_2_S system, the Li ions have liquid-like mobility [Fig. 1b]. The fluidization of Li ions brings the contribution of Li atom convection and modifies the energy exchange behavior between Li and S ions. Besides, the flow of Li ions will also make the phonon scattering more complex than that occurring in the pure solid state.

### Temperature dependent thermal conductivity of various heat carriers

**Fig. 2:**
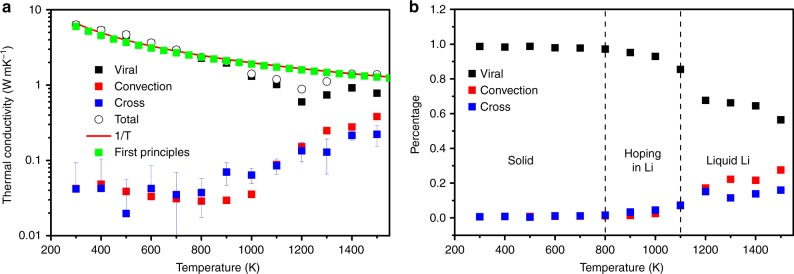
Contributions to temperature dependent thermal conductivity. **a** Total thermal conductivity (TC) and its contributions from the virial, the convection and the cross terms computed using Green-Kubo equilibrium molecular dynamics (GK-EMD) simulations as well as the comparison of the total TC obtained from GK-EMD and Boltzmann transport equation (BTE); **b** TC contribution % from the corresponding terms as a function of temperature

To quantitatively analyze the thermal transport behaviors in Li_2_S, we decompose the total TC into the contributions from phonons, convection, and phonon-convection interactions. We start from the expression of the heat current $\vec Q$ that is used to calculate the thermal conductivity in GK-EMD^23^:

1 $$\vec Q = \mathop {\sum}\limits_i^N {\kern 1pt} e_i\left( {\vec v_i - \vec v} \right) + \frac{1}{2}{\kern 1pt} \mathop {\sum}\limits_{i = 1} {\kern 1pt} \mathop {\sum}\limits_{j = 1;j \ne i}^N {\kern 1pt} \left[ {\vec F_{ij} \cdot \left( {\vec v_i - \vec v} \right)} \right]{\kern 1pt} \vec r_{ij},$$

in which, *e*_*i*_, $\vec v_i$, and $\vec v$ are the energy, velocity of atom *i* and barycentric velocity of the system, respectively. $\vec r_{ij}$ and $\vec F_{ij}$ are position and force vectors between atoms *i* and *j*, respectively. The first term in Eq. (1) results from the convection of particles, which accounts for the heat carried by ions convection. The last term is caused by interactions between atoms, i.e. atomic vibrations, which include phonons and diffusons. Based on the obtained convectional and virial heat flux, we can decompose the total TC into the contributions from the convection of liquid Li ions, denoted as *κ*_convection_, virial inter-atomic vibrations, i.e., lattice vibrations, referred to as *κ*_virial_, and the term of the lattice-convection interactions *κ*_cross_^13^. *κ*_cross_ is resulted from the cross correlation between the heat carried by the lattice and by the moving ions. It is worth to note that *κ*_cross_ is different from the phonon-liquid scattering. The later one is actually included in *κ*_virial_. To varify that, we consider a system where Li ions are fixed while all other conditions being identical to the ones of the original Li_2_S system. Such a system indeed yields a larger virial thermal conductivity. The *κ*_virial_ difference of the two systems is generated by the phonon-liquid scattering. Apart from this scattering, in the realistic system, heat can be transferred between moving Li ions and lattice vibration, which is characterized by *κ*_cross_. The phenomenological difference between phonon-liquid scattering and *κ*_cross_ is as follows: phonon-liquid scattering preserves the energy in solid lattice while *κ*_cross_ exchanges energy between solid and liquid. Figure 2 illustrates the temperature dependent thermal conductivity of Li_2_S and the contributions from different parts obtained by GK-EMD. In accordance with the properties of diffusion coefficient, the temperature dependent TC can also be divided into three regimes: (i) Below 800 K, where both S and Li ions vibrate around their equilibrium positions, phonons are the dominant heat carriers in Li_2_S and the contributions to TC from the convection and cross terms can be ignored as expected [Fig. 2]. In this temperature range, the temperature dependent TC can be well fitted with the 1/*T* power law, which is a typical feature of the Umklapp phonon–phonon scattering process^24^. (ii) In the temperature range from 800 to 1000 K, the virial part, which can be treated as the contribution of propagnons since Li_2_S is a solid, is still the main contributor to the total TC (>90% as shown in Fig. 2b). However, the cross and convection parts start to play non-negligable roles with the cross-term being more significant. This is due to the fact that the hopping of Li ions to the positions of S ions can scatter phonons dramatically (much stronger than the intrinsic phonon–phonon scattering) as it breaks the periodicity of the system. As a consequence, the energy exchange between phonons and flowing Li ions becomes more efficient which is also the reason why the virial term decreases faster than 1/*T* in this temperature range [Fig. 2a]. In addition, recent experiments^6^ also show that the large movement of liquid ions strongly increases the anharmonicity of the PCPLS, which supports the argument that phonons can be scattered by the large movement of liquid ions in the system. Since only a small portion of Li ions hop to other sites (see detailed analysis below), the convection term is still small compared to the virial term. (iii) When *T* ≥ 1100 K, due to the fluidization of Li ions, the TCs of the convection term *κ*_convection_ and the cross term *κ*_cross_ increase rapidly, leading to significant contributions to the total TC [Fig. 2b]. Meanwhile, we find that the virial term *κ*_virial_ follows a dip in the vicinity of 1200 K, above which *κ*_virial_ increases with temperature [Fig. 2a]. This is yielded by the extremely short mean free path (MFP) vibrations, comparable to diffusons. The contribution of diffusons, which has been proven to be one of the main heat carriers in liquid^25^, to the total TC is increasing with temperature (see detailed explanations below).

To validate our results obtained from ReaxFF MD, we compare the phonon dispersion results from experimental results^26^, as well as ab initio and ReaxFF MD calculations (Supplementary Figs. 1 and 2) and a satisfactory agreement was obtained. Actually, the adopted ReaxFF can capture the experimental voltage profile during lithiation of sulfur cathode^10^ as demonstrated by Islam et al. To compare our results with static calculations, we also computed the lattice TC by solving the BTE with the 2nd and 3rd force constants evaluated from first-principles calculations when the Li_2_S is regarded as *β*-phase in the entire temperature range, and its comparison with the GK-EMD results is shown in Fig. 2a. It is interesting to note that the ab initio BTE results agree reasonably well with those calculated from the ReaxFF MD below 800 K, where Li_2_S is in complete solid state. The above comparisons indicate that our ReaxFF modeling can provide correct and qualitative trends of heat transfer properties of PCPLS. It is worth noting that the TC from ab initio BTE follows the 1/*T* trend in the entire temperature range, which is related to the fact that ab initio BTE relies on the hypothesis that the lattice remains unaffected at any temperatures and does not take into account the phase transition effect. As a result, the ab initio BTE method is not applicable to study the thermal transport in the materials undergoing phase transition, where MD with ReaxFF approach has to be applied for such investigations. It is worth mentioning that our TC obtained from ab initio BTE is lower than that in ref. ^27^, which may be generated by the different calculation conditions (e.g., exchange correlation functionals, K-point sampling, softwares, etc).

### Trajectories of ions at different temperatures

**Fig. 3:**
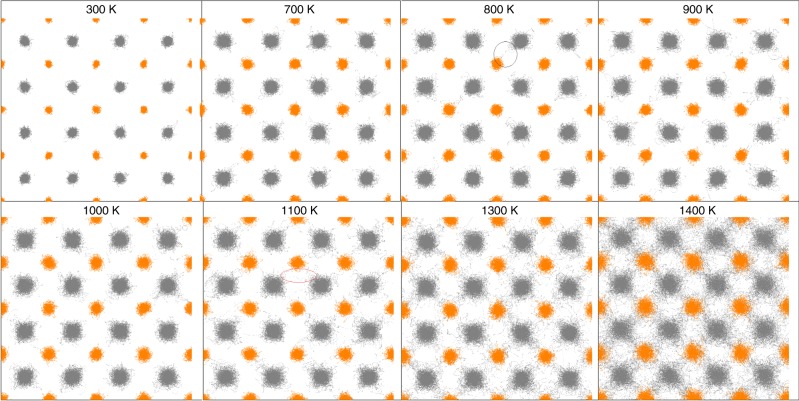
The trajectory of ions in Li_2_S at different temperatures. Vibration trajectories of Li (gray) and S (orange) ions at various temperatures from ab initio molecular dynamics simulations

To deeper understand the temperature behavior of TC, we trace the time trajectories of Li and S ions at different temperatures and project them onto the {100} lattice plane [Fig. 3]. Therefore, Fig. 3 is a superposition of many frames at different times, and the trajectory represents the range that the atoms travel in space. It can be observed that at low temperatures (*T* < 800 K), both Li and S ions vibrate around their equilibrium positions with small amplitudes, following the feature of a crystal structure. In this case, the TC should be fully contributed from phonons, i.e., the *κ*_virial_ part. In the medium temperature range from 800 to 1000 K, although Li_2_S can still be treated as a crystal, the Li ions have the possibility to hop to the neighboring S sites as shown in Fig. 3, where the hopping of Li ions to the S sites is indicated by the black circles. When the Li ions hop to the S site, the S ions will be pushed away a little bit due to the repulsive interactions between neighbors. This is a sign of the enlarged trajectory distribution for S atoms compared to that at lower temperatures. The solid state of Li_2_S in this temperature range is also confirmed by the calculated diffusion coefficients [Fig. 1b]. The hopping of Li ions to S sites scatters phonon much more strongly than the normal anharmonic scattering does, leading to a more notable decrease of TC than the one of 1/*T*. Due to the decrease of phonon TC, the relative TC contributions from *κ*_convection_ and *κ*_cross_ increase to 2% and 4%, respectively. When the temperature is higher than 1100 K, Li ions can vibrate away from their equilibrium positions and flow through the S sublattices by hopping between neighboring Li atoms, the rate of which increases with temperature (red circles in Fig. 3). This phenomenon is corroborated by the estimated diffusivity of Li and S ions: the diffusivity of Li ions is increased by more than one order of magnitude from 1000 to 1100 K while that of S ions only has an incremental increase. Both results indicate that Li ions fluidize beyond 1000 K and Li_2_S becomes a PCPLS. Due to the fluidization of Li ions, the contribution to the total thermal conductivity of the liquid related term *κ*_convection_ is enhanced significantly (from about 10% to around 40%). In addition, we note that the temperature dependent virial term (phonon term) follows a dip around 1200 K, beyond which it increases with temperature after a fast decrease below 1200 K. This phenomenon may arise from the vibration modes with extremely short MFPs, whose contribution to the total TC increases with temperature. In fact, the diffusion of Li ions will leads to their collision with the S sublattices, thus strongly scatters phonons in the S sublattices. Besides, at such high temperatures (>1200 K), the phonon–phonon interaction is also extremely strong. Both reasons indicate that propagons in the S sublattices almost do not carry heat. On the other hand, it is well known that apart from the wave-like phonon transport mechanism (i.e., propagons) in crystals^28^, the heat can also be carried by lattice vibrations with MFPs shorter than the nearest neighbor atoms via hopping of forces. Hopping events become more efficient at high temperatures due to the increased atomic vibrational amplitude. As a result, the presence of the dip in the evolution of the virial TC is due to the interplay between the enhanced scattering rate and the enhanced extremely short MFP vibration transport. The quantitative contribution from the extremely short MFP vibrations can be calculated by FDDDM and is reported below.

### Characterize four different heat carriers based on mean free path analysis

**Fig. 4:**
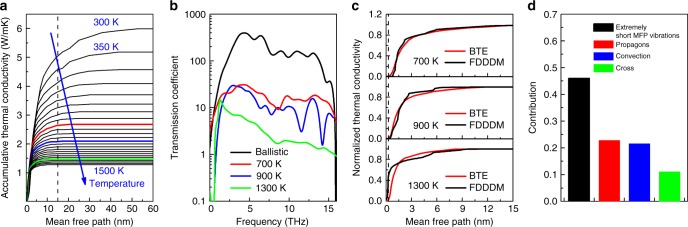
Characterize four different heat carriers based on the mean free path (MFP) analysis. **a** Accumulative thermal conductivity versus MFP at different temperatures from first principles calculations. **b** Frequency dependent transmission coefficient computed via Eq. (3). **c** Normalized accumulative thermal conductivity versus MFP from first principles, i.e., Boltzmann transport equation (BTE), and frequency-dependent direct decomposed method (FDDDM) (reactive force filed molecular dynamics) simulations. The black dashed line indicates the distance between the nearest neighbors. **d** Comparison of the relative contributions of different heat carriers in Li_2_S at 1300 K

To quantify the frequency resolved fluidization effect of Li ions, we also calculated the phonon MFP and transmission coefficient using FDDDM^15,17^. It should be noted that although the Li ions can diffuse across the S sublattice, there is no net Li flow from one thermostat to another during the NEMD simulations. This is proved by our spatial Li density distribution in Supplementary Fig. 3a, which shows that the Li density along the temperature gradient direction is unchanged. Besides, the unchanged convection thermal conductivity shown in Supplementary Fig. 3b, when the temperature difference is reduced from 80 to 60 and 40 K, and the radial distribution function of Li_2_S at 300 and 1300 K (Supplementary Fig. 3c), further proves there is no net Li flow during NEMD simulations. From FDDDM, the heat current spectrum *Q*(*ω*) can be obtained via Eq. (2)

2 $$Q(\omega ) = \mathop {\sum}\limits_{i \in L} {\kern 1pt} \mathop {\sum}\limits_{j \in R} {\kern 1pt} {\mathrm{Re}}{\kern 1pt} \left[ {\int_{ - \infty }^{ + \infty } {\kern 1pt} \left\langle {\left. {{\textstyle{{\partial U_j} \over {\partial \vec r_{ji}}}}} \right|_\tau \vec v_i(0) - \left. {{\textstyle{{\partial U_i} \over {\partial \vec r_{ij}}}}} \right|_\tau \vec v_j(0)} \right\rangle {\kern 1pt} e^{i\omega \tau }\mathrm{d}\tau } \right],$$

in which *U* refers to the interatomic potential energy, $\vec v$ and $\vec r$ denote the velocity and position of atoms, respectively. Combining the Landauer theory and Eq. (2), we have

3 $$\begin{array}{*{20}{l}} {T(\omega )} \hfill & = \hfill & {{\textstyle{{Q(\omega )} \over {k_b{\mathrm{\Delta }}T}}}} \hfill \\ {} \hfill & = \hfill & {{\textstyle{1 \over {k_b{\mathrm{\Delta }}T}}}{\kern 1pt} \mathop {\sum}\limits_{i \in L} {\kern 1pt} \mathop {\sum}\limits_{j \in R} {\kern 1pt} {\mathrm{Re}}{\kern 1pt} \left[ {{\int}_{ - \infty }^{ + \infty } {\kern 1pt} \left\langle {\left. {{\textstyle{{\partial U_j} \over {\partial \vec r_{ji}}}}} \right|_\tau \vec v_i(0) - \left. {{\textstyle{{\partial U_i} \over {\partial \vec r_{ij}}}}} \right|_\tau \vec v_j(0)} \right\rangle {\kern 1pt} e^{i\omega \tau }\mathrm{d}\tau } \right],} \hfill \end{array}$$

where Δ*T* is the temperature difference between the left and the right leads^16^. Obviously, the total heat flux Q depends on the cross-section of the sample, which arises from the band folding effect. To evaluate the MFPs, we have adopted the same cross-section as of 4 × 4 unit cells for all the transmission estimations. Once the transmission function is obtained, we can calculate the MFP Λ(*ω*) via Λ(*ω*) = *T*(*ω*)*L*/[*M*(*ω*) − *T*(*ω*)]^29^, in which *L* and *M*(*ω*) are the length of the system and the ballistic transmission coefficient, respectively. Three typical temperatures (700, 900, and 1300 K) are chosen for analysis. Here *L* = 20 nm is chosen for FDDDM calculations, which is long enough to eliminate the size effect as the typical MFP is between 10 and 15 nm at the indicated temperatures as shown by our ab initio BTE results [Fig. 4a]. The details for the calculation of *M*(*ω*) are given in Methods section. Figure 4b reports the transmission coefficients of Li_2_S (700 and 900 K) and of the S sublattices (1300 K). As expected, the transmission coefficient reduces on average with temperature as lattice vibrations are more strongly scattered by liquid and themselves at high temperatures. To facilitate the comparisons between the MFPs computed using FDDDM and BTE, we plotted the accumulative TCs based on the MFPs obtained from both FDDDM and BTE in Fig. 4c. At 700 K, the accumulative TCs from both methods fit exactly, which again validates the accuracy of our applied ReaxFF model in MD simulations. When the temperature rises to 900 K, the system is in its crystal state as discussed above. Consequently, the accumulative TC calculated by BTE and FDDDM are still comparable. The difference in the long MFP region—long MFP phonons contribute less to TC in FDDDM than that in BTE—is due to the fact that phonons are scattered by the hopping of a few Li ions [Fig. 3]. Finally, with temperature increasing to 1300 K, the effective MFPs of lattice vibrations in FDDDM, naturally including all phonons, are short due to the strong scattering. It is also remarkable that, the vibrations with effective MFPs smaller than 0.265 nm, i.e., the distance between the nearest neighbors, contribute around 66% to the virial TC. This is due to the fact that large amplitude vibrations at high temperatures dramatically enhance the hopping force between neighboring atoms, thus increase the heat carried by diffusons. At 1300 K, diffusons contribute up to 46% to the total TC as revealed in [Fig. 4d], which is calculated from the total heat current of the S sublattice, i.e., the summation of the heat fluxes of all S ions. As a consequence, *κ*_virial_ will increase with temperature when *T* ≥ 1200 K as we observe in Fig. 2a. On another hand, we also find that the virial contribution decreases again at 1400 K. The temperature dependence of the virial term at high temperature range *T* ≥ 1200 K results from two competing heat carries (propagons vs. diffusons). When diffusons are dominant, the virial term increases with temperature firstly but eventually reaches a plateau. Therefore, the virial term decreases again due to the negative temperature dependence of the phonon contribution.

## Discussion

Here we have to emphasize that although the lattice vibrations with extremely short mean free path in our work show some similarities to the diffusons as defined in disordered solid systems, they are actually different from each other. It is well known that diffusons defined by Allen et al.^28^ transport energy via lattice vibrations. While in our case, the Li ions can diffuse in the S sublattice. Thus it is not lattice vibrations any more and the modes with extremely short mean free path is due to the collision between the diffuse Li ions and the S sublattices. Phenomenologically, since all the vibrational modes are populated (Boltzmann distribution) in MD simulations, the MD simulated diffuson thermal conductivity is temperature independent. Meanwhile, it is well known that the thermal conductivity of propagons in disordered solid systems is also temperature independent since anharmonicity is much less important compared to the disorder scattering^30^. Therefore, the lattice thermal conductivity in disordered solid systems is temperature independent, which is confirmed by Moon et al.^30^ However, in our MD results, we found that the lattice thermal conductivity shows a strong temperature dependence when *T* > 1200 K, which is different from the solid disordered systems. In terms of mechanism, on one hand, the thermal conductivity of propagons (in the S sublattices) in our PCPL system decreases with temperature due to the enhanced anharmonicity. On the other side, increasing temperature will make Li ions collide with S ions more frequently due to the enhanced diffusivity, which can increase the thermal conductivity contributed from those vibrational modes with extremely short mean free path. With the further increase of temperature, the collision frequency between Li ions and S ions will reach a plateau, which is confirmed by our diffusion coefficient result. From Fig. 1b, we know that the diffusion constant of Li ions above 1100 K increases dramatically with temperature until 1400 K, after which the diffusion coefficient is saturated. The saturation of diffusion coefficient of Li ions finally leads to the saturation of thermal conductivity contributed by lattice vibrations with extremely short mean free path. As a result, the interplay between the thermal conductivity contributed by diffusons and propagons results in the observed temperature dependent thermal conductivity.

In conclusion, we have performed ReaxFF molecular dynamics and first-principles simulations of the thermal transport mechanisms in Li_2_S from 300 to 1500 K, and quantified the respective contributions of TC from lattice vibrations, convection, and lattice-convection interaction. Below 800 K, the system can be treated as an ideal crystal where the heat is fully carried by phonons and the temperature dependent TC follows the traditional 1/*T* relationship. In the medium temperature range (800–1000 K), although Li_2_S can be still treated as a crystal in general, the hopping of a few Li ions cause a non-negligible contribution of *κ*_convection_ and *κ*_cross_, and also lead to the deviation of the crystal TC away from the 1/*T* relationship. Above 1100 K, Li ions become liquid-like and the contributions of *κ*_convection_ and *κ*_cross_ increase significantly. Furthermore, there is an interplay between the enhanced phonon scattering and the increased force hopping between neighboring atoms as temperature arises, which results in a dip in the evolution of the virial term in the vicinity of 1200 K. When the temperature is higher than 1200 K, the virial TC increases with temperature due to the contribution of vibrations with extremely short MFP (i.e., diffusons). This point is validated by the evolution of the accumulative TC with MFP. At 1300 K, more than 46% of the heat carried by the S sublattices is contributed by the carriers with MFP smaller than a few angstroms, which is the typical hopping distance. Our study provides a clear physical map of the heat transport in phase-change materials and describes the key mechanisms to guide the design of the future thermoelectric materials and battery electrodes.

## Methods

### First-principles calculations

All the first-principles simulations are computed using Vienna Ab initio Simulation Package (VASP) based on density functional theory (DFT). We use the pseudopotential with generalized gradient approximation (GGA), parametrized by Perdew–Burke–Ernzerhof (PBE)^31^ for the exchange-correlation functional. Periodic boundary conditions are applied in all three directions. A plane basis with cutoff energy of 650 eV for Li_2_S and the Monkhorst-Pack scheme^32^ is used to generate an 8 × 8 × 8 k-point mesh. Before any electrostatic potential or interatomic force constant calculation, the atomic structures and cell size are fully relaxed until the energy difference and the Hellman–Feynman force are converged within 1 × 10^−8^ eV and 1 × 10^−7^ eV/Å, respectively. Then, we run Born-Oppenheimer molecular dynamics (BOMD) of the 2 × 2 × 2 supercell for 15 ps to obtain the atomic trajectory for the mean square displacement (MSD) calculation^9^, in which a timestep of 3 fs is chosen for Li_2_S and the k-point mesh is shifted to gamma point. In the BOMD simulations, the convergence criterion for energy is 5 × 10^−4^ eV. The diffusion coefficient is obtained by fitting the MSD curve. The lattice parameter is 0.570 nm which is in good accordance with the experimental value of 0.571 nm. The phonon dispersion and phonon density of state are shown in Supplementary Figs 1 and 2, as compared with our ReaxFF molecular dynamics (MD) results computed using spectral energy density (SED)^33^. The details of SED is given below.

### Molecular dynamics simulations

All the classical MD simulations are performed with the LAMMPS package^34^. We first run 500 ps with timestep of 0.25 fs to reach the target temperature using NVT (constant particles, volume and temperature) ensemble. Then, for the equilibrium molecular dynamics simulations, we run 1 ns with NVE (constant particles, volume, and energy) ensemble to generate the instant heat current that is used to calculate the thermal conductivity *κ* via Green-Kubo theory^13^. For each case 30 independent runs are performed in order to obtain a stable averaged *κ*. The correlation time considered in our simulation is 20 ps, which is long enough to obtain the converged thermal conductivity. We choose 4 × 4 × 4 unit cell box to calculate the thermal conductivity, which has eliminated the size effect in GK-EMD (Supplementary Fig. 4). To achieve a high enough resolution of the spectral energy density (SED) contour while keeping acceptable model size and computational time, we use a 2 × 1000 × 2 supercell to perform the SED calculations, and output the atomic velocities every 25 fs for a total sampling time of 50 ps. The sound velocity is calculated as 4719.43 m/s [(3453.24 × 2 + 7251.81)/3] for ReaxFF MD simulations and 10,059.72 m/s [(11,277.13 + 7275.08 + 11,626.94)/3] for first-principles calculations, respectively. Although, the ReaxFF MD underestimate sound velocity strongly comparing to first principle results, the average group velocity for the acoustic phonon braches are approaching [3813.04 m/s for ReaxFF MD vs. 3729.15 m/s for first principle]. The agreement of average group velocity in the low temperature limit assures the ReaxFF can qualitatively predict the right phenomenon. At the high temperature (1200 K in Supplementary Fig. 2), the acoustic phonon branches are shifting down due to softening effect caused by the increased temperature (i.e., renormalization of phonon dispersion). Here, we did not consider the temperature effects in our first-principles calculations of phonon dispersions, which could be one of the reasons for the big difference between first-principles and ReaxFF results of phonon dispersions. Nevertheless, this difference would not change the trend and the qualitative conclusion in our main text. Meanwhile, first-principles calculations give a little bit different result comparing to ReaxFF MD computations in the high frequency region (Supplementary Fig. 1). Such a difference may lead to the inaccurate thermal conductivity value of superionic *α*-phase Li_2_S; however, the mechanism which is the main topic in this manuscript will not be changed. The SED of superionic *α*-phase Li_2_S at 1200 K is also plotted in Supplementary Fig. 2. It is clear to find that phonons also exist in liquid Li (Supplementary Fig. 2c) ions due to the interactions between Li and S (or Li) ions, which is in accordance with the previous consequence^25^.

For non-equilibrium molecular dynamics (NEMD) simulations, fixed boundary conditions are applied to both ends of the system. Near to the fixed boundaries, a hot and cold reservoir with a temperature difference of 80 K is applied such that a temperature gradient is established along the concerned direction (*x*-direction in our simulations) after running 2 ns. In the last 500 ps NEMD run, we output the trajectories of atomic velocities every five time steps for the transmission coefficient calculation [Eq. (3)]. To calculate the ballistic transmission coefficient *M*(*ω*), we choose the length of the model to be short enough (only 0.8 nm) to make sure all the phonons are in the ballistic transport region. In NEMD simulations, a temperature gradient is applied, the convection of Li ions in S sublattices might lead to a net flow of Li ions from the hot thermostat to the cold one. To check that, we calculated the number of Li ions in the 11.5–13.0 nm region averaged with time (Supplementary Fig. 3a). It can be seen that the number of Li ions in the concerned regions almost keep constant, which indicates that there is no net Li flow in our NEMD simulations. To further prove that, we also reduced the temperature difference between the two thermostats from 80 to 60 and to 40 K in NEMD simulations (the system temperature is 1300 K). If the temperature difference can drive a Li ion density difference, a different temperature gradient would result in a different convection thermal conductivity. However, as can be found in Supplementary Fig. 3b, the convection thermal conductivity is almost a constant, which once again confirms the temperature difference applied in our NEMD simulations will not build-up an internal electrical field between the two thermostats. Finally, if there is a flow of Li ions from the hot part to the cold part, the driving force should be the density difference at different temperatures. While from the radial distribution function of Li_2_S at 300 and 1300 K (Supplementary Fig. 3c), we concluded that the averaged nearest neighbor distances are almost the same at the two temperatures, which means the density of Li ions is the same. In our NEMD simulations the temperature difference is only 80 K and is much smaller than 1000 K (1300–300 K). As a result, the density of Li ions should be the same in the two thermostats and no net Li ion flow will happen.

The computational time in ReaxFF MD and first-principles calculations is also compared in Supplementary note 1 (Supplementary Fig. 5).

### Boltzmann transport equations

The BTE, which is formulated by Peierls^35^, can be solved by assuming the scattering term expanded to its first-order perturbation. Without considering the impurities and boundaries, the linearized BTE can be recasted into^14,36,37^

4 $$\begin{array}{*{20}{l}} { - c\left( {\vec k,\nu } \right)\,\left( {{\textstyle{{\partial \bar n\left( {\vec k,\nu } \right)} \over {\partial T}}}} \right)} \hfill & = \hfill & {\mathop {\sum}\limits_{\left( {\vec k\prime ,\nu \prime } \right),\,\left( {\vec k\prime\prime ,\nu \prime\prime } \right)} {\kern 1pt} \left[ {{\mathrm{\Gamma }}_{\left( {\vec k\prime ,\nu \prime } \right),\,\left( {\vec k\prime\prime ,\nu \prime\prime } \right)}^{\left( {\vec k,\nu } \right)}{\kern 1pt} \left( {f_{\left( {\vec k,\nu } \right)}^1 + f_{\left( {\vec k\prime ,\nu \prime } \right)}^1 - f_{\left( {\vec k\prime\prime ,\nu \prime\prime } \right)}^1} \right)} \right.} \hfill \\ {} \hfill & {} \hfill & { + \frac{1}{2}\left. {{\mathrm{\Gamma }}_{\left( {\vec k,\nu } \right)}^{\left( {\vec k\prime ,\nu \prime } \right),\,\left( {\vec k\prime\prime ,\nu \prime\prime } \right)}\left( {f_{\left( {\vec k,\nu } \right)}^1 - f_{\left( {\vec k\prime ,\nu \prime } \right)}^1 - f_{\left( {\vec k\prime\prime ,\nu \prime\prime } \right)}^1} \right)} \right]} \hfill \end{array},$$

where $\left( {\vec k,\nu } \right)$ is the phonon mode with wave vector $\vec k$ and branch *ν*, *c*, $\bar n$, *f*, and *T* are the specific heat capacity, equilibrium phonon population, first-order perturbation and system temperature, respectively. Γ is the scattering rate at equilibrium of a process where a phonon mode is scattered by absorbing another phonon to generate the third phonon or decayed to two different phonons. The scattering rate matrix can be obtained based on the Fermi s golden rule. As phonons are bosons, they follow the Bose–Einstein distributions, i.e., $\bar n\left( {\vec k,\nu } \right) = 1{\mathrm{/}}\left( {e^{\hbar \omega \left( {\vec k,\nu } \right){\mathrm{/}}k_BT} - 1} \right)$, where *ħ* and $\omega \left( {\vec k,\nu } \right)$ are the reduced Planck constant and phonon frequency, respectively. Together with the Fourier's law, Eq. (4) can be used to calculate the thermal conductivity *κ*_ab initio_ of a solid without phase transition. In the BTE, the 2 × 2 × 2 supercell with 96 atoms is used to generate the second and third-order force constants which will be used as the input of the Boltzmann transport equation (BTE) solver^14^. We have considered the third-order interaction force constants up to the eighth shell of neighbors in a 2 × 2 × 2 supercell with 3 × 3 × 3 k-point mesh. Convergence tests are reported in Supplementary Fig. 6. Based on our results, we choose 13 × 13 × 13 and 8th neighbor in our calculations. What is worth noting is that, the fourth order scattering is important when there is big band gap between the acoustic and optical phonon branches (normally for the high thermal conductivity materials), where the three-phonon process is prohibited^38^. While for the low thermal conductivity material (i.e., Li_2_S), because the phonon branches are quite dense (see Supplementary Fig. 1), the contribution to the thermal conductivity from three-phonon process is much larger than that from the possible four-phonon scattering process. Generally speaking, the scattering matrix for four-phonon scattering is much smaller than for three-phonon scattering process. Therefore, for the material with such a low thermal conductivity (lower than 10 W mK^−1^), the fourth order phonon scattering process can be safely ignored. This fact is largely recognized in thermal transport community.

## Electronic supplementary material

Supplementary Information

## Data Availability

The data that support the findings of this study are available from the corresponding author upon request.
