# Author Correction: Thermal transport crossover from crystalline to partial-crystalline partial-liquid state

**DOI:** 10.1038/s41467-018-07749-y

**Published:** 2018-12-11

**Authors:** Yanguang Zhou, Shiyun Xiong, Xiaoliang Zhang, Sebastian Volz, Ming Hu

**Affiliations:** 1 Aachen Institute for Advanced Study in Computational Engineering Science (AICES), RWTH Aachen University, Aachen 52062, Germany; 2 Department of Mechanical and Aerospace Engineering, University of California Los Angeles, Los Angeles, CA 90095 USA; 3 Functional Nano and Soft Materials Laboratory (FUNSOM) and Collaborative Innovation Center of Suzhou Nano Science and Technology, Soochow University, Suzhou 215123, China; 4 Joint International Research Laboratory of Carbon-Based Functional Materials and Devices, Soochow University, Suzhou 215123, China; 5 Key Laboratory of Ocean Energy Utilization and Energy Conservation of Ministry of Education, School of Energy and Power Engineering, Dalian University of Technology, Dalian 116024, China; 6 CNRS, UPR 288 Laboratoire d’Energétique Moléculaire et Macroscopique, Combustion (EM2C), Ecole Centrale Paris, Grande Voie des Vignes, 92295 Châtenay-Malabry, France; 7 LIMMS/CNRS-IIS(UMI2820) Institute of Industrial Science, University of Tokyo 4-6-1 Komaba, Meguro-ku Tokyo, 153-8505 Japan; 8 Institute of Mineral Engineering, Faculty of Geosciences and Materials Engineering, RWTH Aachen University, Aachen 52064, Germany; 9 Department of Mechanical Engineering, University of South Carolina, Columbia, SC 29208 USA

Correction to: *Nature Communications* ; 10.1038/s41467-018-07027-x; published online 9 November 2018.

The original version of this Article incorrectly omitted an affiliation of Sebastian Volz: ‘LIMMS/CNRS-IIS(UMI2820) Institute of Industrial Science, University of Tokyo 4-6-1 Komaba, Meguro-ku Tokyo 153-8505 JAPAN’. This has now been corrected in both the PDF and HTML versions of the Article.

