# Supplementary material for: Thermal transport crossover from crystalline to partial-crystalline partial-liquid state
Source: Nat Commun. 2018 Nov 9;9:4712. doi: 10.1038/s41467-018-07027-x (PMC6226496; doi:10.1038/s41467-018-07027-x)
Supplement: Supplementary file 1 — Supplementary Information [file 41467_2018_7027_MOESM1_ESM.pdf]

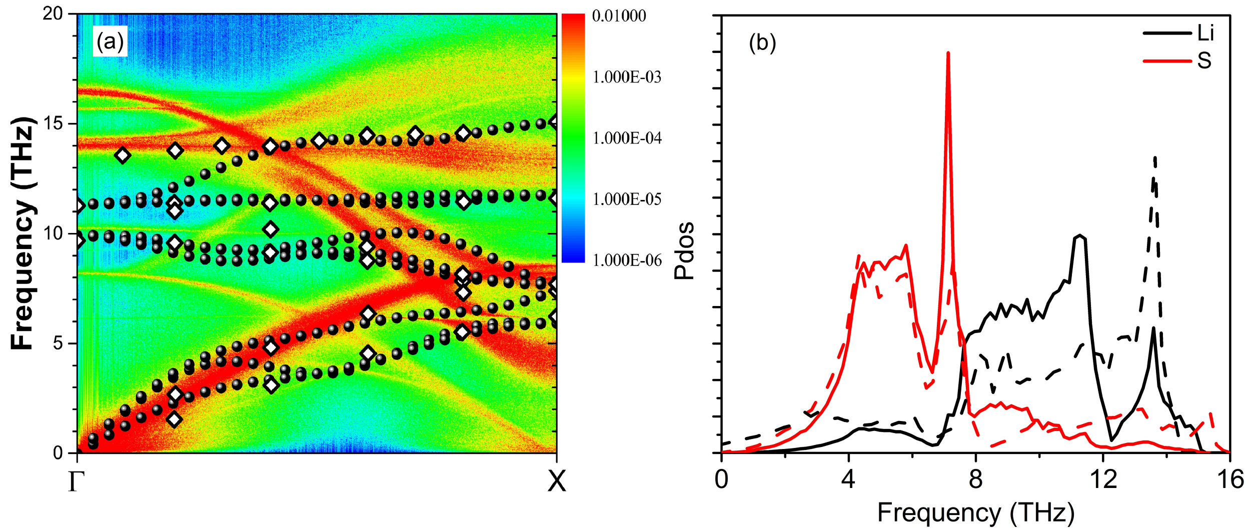

Supplementary Figure 1: (a), Comparison of phonon dispersion calculated by first-principles (black dots) and molecular dynamics (spectral energy density, at 1 K), respectively. (b), the phonon density of state between first-principles (solid lines) and ReaxFF potential (dashed lines) calculations. The diamond-shaped dots are generated from experimental results [1].

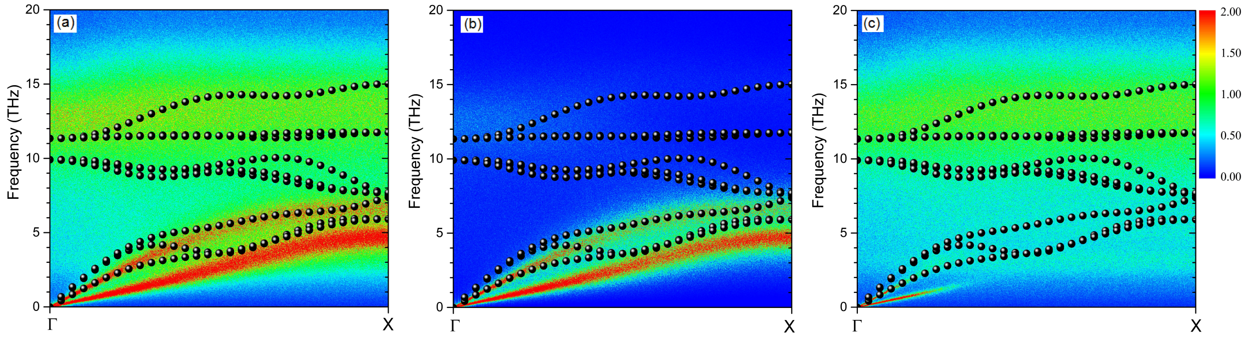

Supplementary Figure 2: Spectral energy density of Li_2S at 1200 K (a), S (b) and Li ions (c). The black dots are first principle results as shown in Supplementary Figure 1.

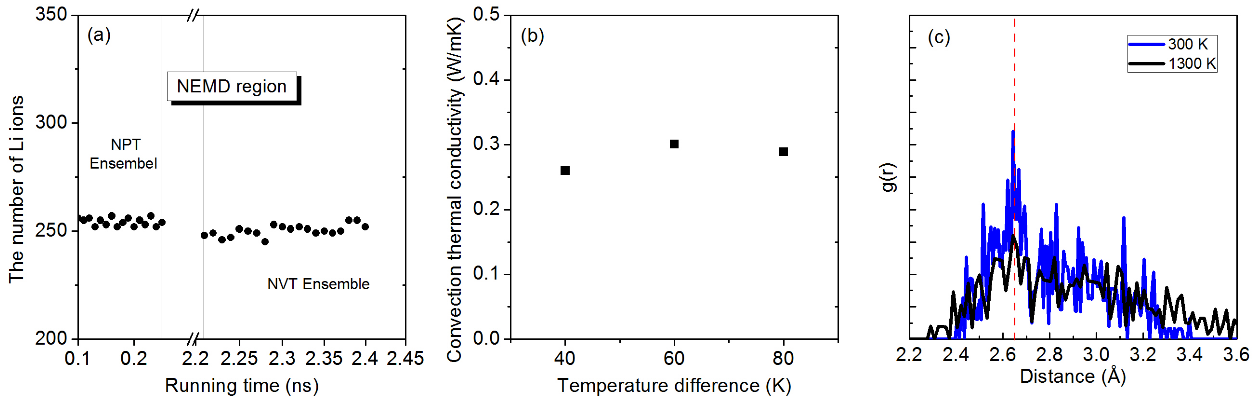

Supplementary Figure 3: (a) The number of Li ions in the 11.5-13.0 nm region in molecular dynamics (MD) simulation (selected simulation segments from structure relaxation and non-equilibrium molecular dynamics (NEMD) simulation); (b) The convection thermal conductivity in NEMD simulations using various temperature differences; (c) Radial distribution function of Li2S at 300 K and 1300 K. The red dashed line stands for the nearest neighbor distance.

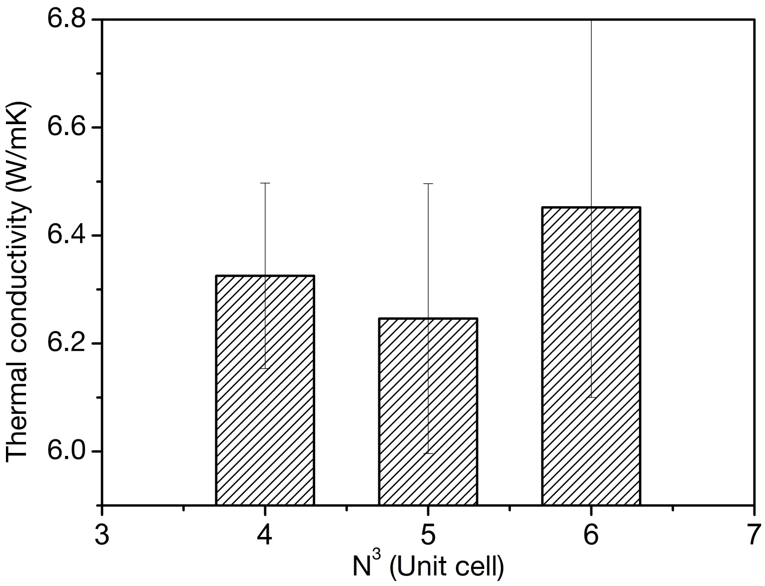

Supplementary Figure 4: Convergence test for thermal conductivity computed by Green-Kubo equilibrium molecular dynamics (GK-EMD).

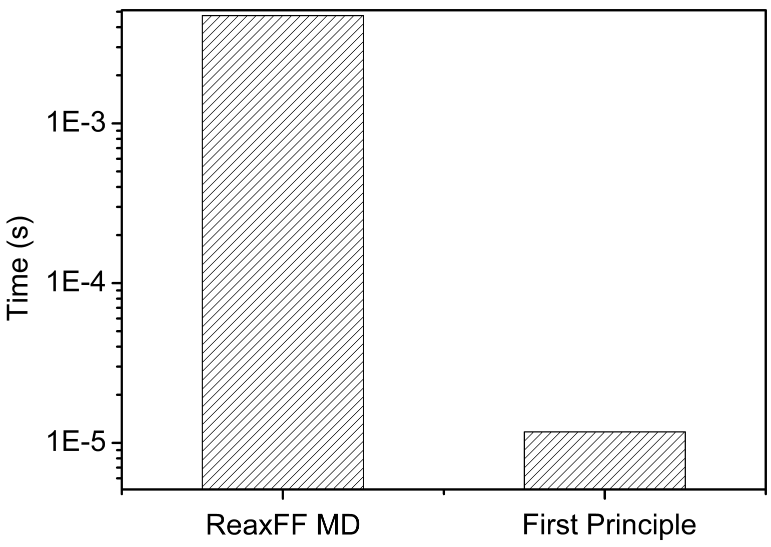

Supplementary Figure 5: The computational time in ReaxFF molecular dynamics (MD) and first-principles calculations.

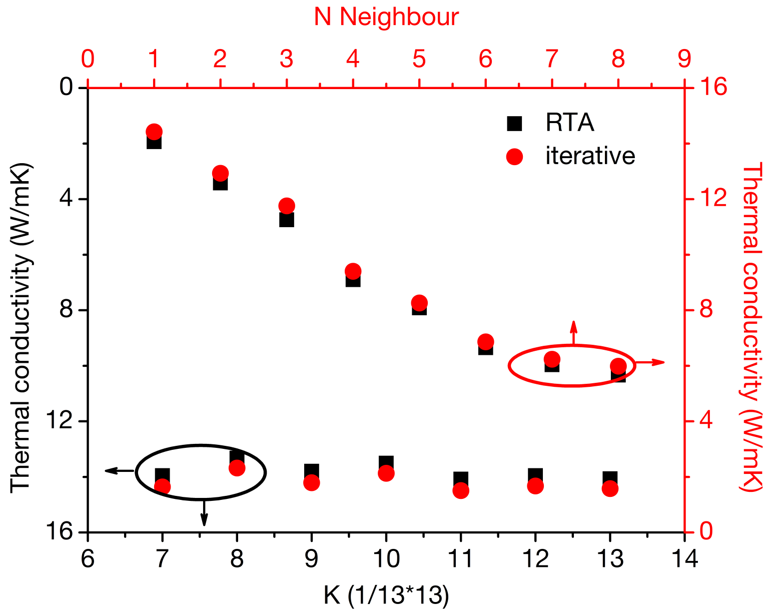

Supplementary Figure 6: Convergence test for third-order interaction force constants used in Boltzmann transport equation (BTE). For the K point test, we choose the 1st Neighbour.

Supplementary Note 1: Relative computational speed of AIMD and ReaxFF MD

The computational time in ReaxFF molecular dynamics (MD) and first-principles calculations is calculated via $Time = t_{total}/(N_{atoms}N_{pro}N_{timestep})$, in which t_{total}, N_{atoms}, N_{pro} and $N_{timestep}$ are the running time, number of atoms, number of processors and number of the total running. Our results (Supplementary Figure 5) show that the ReaxFF MD is much faster than the First principle calculations (about 300 times), which indicates that the computational inexpensive method ReaxFF MD is appropriate to study the properties of Li_2S.

Supplementary References:

1. Buehrer, W., Altorfer, F., Mesot, J., Bill, H., Carron, P. & Smith, H. Lattice dynamics and the diffuse phase transition of lithium sulphide investigated by coherent neutron scattering. *J. Phys. Cond. Mat.* **3**, 1055 (1991).
